# Sensitivity of *Ixodes ricinus* (L., 1758) and *Dermacentor reticulatus* (Fabr., 1794) ticks to entomopathogenic fungi isolates: preliminary study

**DOI:** 10.1007/s00436-020-06805-1

**Published:** 2020-07-14

**Authors:** Anna Szczepańska, Dorota Kiewra, Kinga Plewa-Tutaj, Dagmara Dyczko, Katarzyna Guz-Regner

**Affiliations:** 1 Department of Microbial Ecology and Environmental Protection, Institute of Genetics and Microbiology, University of Wroclaw, Przybyszewskiego str. 63/77, 51-148 Wroclaw, Poland; 2 Department of Microbiology, Institute of Genetics and Microbiology, University of Wroclaw, Przybyszewskiego str. 63/77, 51-148 Wroclaw, Poland

**Keywords:** *Ixodes ricinus*, *Dermacentor reticulatus*, Entomopathogenic fungi, Biocontrol

## Abstract

Entomopathogenic fungi of the genus *Beauveria* and *Metarhizium* play an important role in controlling the population of arthropods. However, the data on their effectiveness against ticks focus mainly on species that do not occur in Europe. The aim of the study was to assess the effectiveness of entomopathogenic fungi against two of the most important tick species in Europe: *Ixodes ricinus* and *Dermacentor reticulatus*. In our study, the majority of tested entomopathogenic fungi strains showed potential efficacy against both tick species; however, *D. reticulatus* was less susceptible in comparison to *I. ricinus*. The observed mortality of ticks was up to 100% by using all commercial strains as well as three out of nine of the environmental strains. Among all tested fungi, the most effective against both tick species was environmental strain *Metarhizium anisopliae* LO4(1) with LC_50_ values: 2.6 × 10^3^ cfu/ml–5.7 × 10^5^ cfu/ml. Botanigard proved to be more effective than MET52 with LC_50_ values: 6.8 × 10^3^ cfu/ml–3.3 × 10^6^ cfu/ml. The conducted bioassays indicate the potential possibility of using the environmental isolates of entomopathogenic fungi, as well as commercial strains in control of local populations of *I. ricinus* and *D. reticulatus*; however, the possibility of using them in vivo requires more research.

## Introduction

Ticks are one of the most important parasites due to transmission of pathogens of tick-borne diseases (TBD). For limiting TBD cases, the individual prophylaxis is mainly recommended; however, the control of the tick population in the environment is also desirable. Among the natural factors limiting the populations of arthropods, there are microorganisms, including entomopathogenic fungi. However, the data concerning their effectiveness against ticks are scarce and show that different tick species, even the different developmental stages, differ in sensitivity to the bioagent.

The study on the efficiency of fungi against ticks was focused mainly on the genus *Rhipicephalus* and additional taxa including *Ixodes scapularis*, *Dermacentor variabilis*, and *Amblyomma variegatum* (Onofre et al. 2001; Kirkland et al. 2004; Tuininga et al. 2009; Hedimbi et al. 2011). The data on the potential use of the entomopathogenic fungi against *Ixodes ricinus*, the tick of the greatest medical and veterinary importance in Europe, are limited and cover mainly the study on larvae and nymphs of this species and engorged adults (Hartelt et al. 2008; Alekseev 2011; Wassermann et al. 2016; Pirali-Kheirabadi et al. 2016). So far, there is no published data involving the possibility of using entomopathogenic fungi against *Dermacentor reticulatus*, an important vector of *Babesia canis*. However, it is known that in the natural conditions, entomopathogenic fungi can infect both *I. ricinus* and *D. reticulatus*. Entomopathogenic fungi isolated from these tick species were identified as *Beauveria bassiana*, *Beauveria tenella*, *Lecanicillium lecanii*, *Isaria fumosorosea*, *Isaria farinosa*, *Verticillium aranearum*, *Beauveria brongniartii*, and *Beauveria pseudobassiana* (Samsinakova et al. 1974; Kalsbeek et al. 1995; Munteanu et al. 2014).

The aims of this study were as follows: (1) to obtain environmental fungi strains with potential entomopathogenic properties; (2) to estimate in vitro the effectiveness of entomopathogenic fungi against the most frequently recorded tick species in Europe, i.e., *I. ricinus* and *D. reticulatus*; (3) to compare the effectiveness of environmental isolates with commercial strains.

## Material and methods

### Tick collection for bioassay

*Ixodes ricinus* and *Dermacentor reticulatus* ticks were collected using the standard flagging method in Wroclaw agglomeration (Poland). Collected ticks were placed in plastic tubes and kept in the refrigerator until they were used for the bioassay (max. for 1 week). Only adults of *I. ricinus* and *D. reticulatus* identified according Estrada-Pena et al. (2017) were used for the bioassay.

### The isolation of entomopathogenic fungi

The entomopathogenic fungi were isolated from 38 soil samples, collected from the Osobowicki Forest area (Wroclaw, SW Poland) using the insect bait method (Zimmermann 1986). As per similar properties, larvae of *Tenebrio molitor* were used (Sharma et al. 2018). Ten larvae were placed in each soil sample dampened with distilled water on Petri dishes (incubation at 22 ± 1 °C, 30 days in the darkness) and checked daily. Dead larvae were removed to the sterile weighing bottle with 100 μl of the sterile distilled water on the bottom margin of the bottle to keep the humidity. The larvae were observed daily for visible fungal growth. After the spore production, individual fungi strains were isolated on potato dextrose agar (PDA, Biocorp).

### Fungi identification

Fungi strains were inoculated onto 25 ml of the liquid and sterile Sabouraud dextrose agar and incubated on a shaker for 7 days. The mycelium was harvested by mechanical filtration in a Büchner funnel (Whatman no. 1 filter paper), washed with sterile distilled water, then frozen at 20 °C, and stored until required. The DNA extraction was carried out using the GenoPlast Biochemicals isolation kit. The ITS region was used to amplify the 5.8S rDNA gene of length range between 600 and 700 bp. The ITS region of isolated species was amplified using the universal primer set ITS4 (5′ TCCTCCGCTTATTGATATGC 3′) and ITS5 (5′ GGAAGTAAAAGTCGTAACAAGG 3′) (Pérez-González et al. 2014). Positive amplifications were purified using the DNA purification kit (GenoPlast Biochemicals) and then sent for sequencing (Genomed, Warsaw). The sequencing results were compared with the sequences listed in the National Center for Biotechnology Information (https://blast.ncbi.nlm.nih.gov/Blast.cgi).

### Preparation of a fungal spore suspension

Eight selected environmental soil isolates were used for the bioassay: 5 strains of *Metarhizium anisopliae* LO4(1), LO10(1), LO52(1), LO52(2), LO47(3), 1 strain of *Metarhizium robertsii* LO26(2), 1 strain of *Isaria fumosorosea* LO34(3), 1 strain of *Beauveria bassiana* LO37(1), and additionally one strain *B. bassiana* IGM1 from the microorganism collection of the Institute of Genetics and Microbiology, University of Wroclaw, isolated from a housefly (*Musca domestica*). Bioassays were also carried out using two commercial strains: *M. anisopliae* (MET52 Granular, Lot: 1511MG09) and *B. bassiana* (22WP Botanigard, Borregaard, BioPlant, Lot: 22WP141002). All fungi strains were cultured on PDA medium (22 °C, 3 weeks). Mature colonies were harvested to 0.1% Tween 80 and centrifuged (4000 rpm, 5 min) to separate the spores from the hyphae. The spore concentration was determined using the Fuchs-Rosenthal chamber.

### Sporulation test and bioassay

Before the bioassay, a spore germination test was performed. The 1 ml of prepared suspension was incubated on PDA medium (room temperature, 18 h). The ratio of germinating to non-germinating spores was counted according to formula: %s = (gs / ngs) × 100 (%), where gs—number of germinating spores and ngs—number of non-germinating spores. Strains with > 90% of germinating spores were used in bioassays.

Ten individual unfed ticks separately for each tick species (*D. reticulatus*/*I. ricinus*) and sex (female/male) were used for each fungal dilution and the control (Hartelt et al. 2008; Pirali-Kheirabadi et al. 2016). Ticks were washed in the sterile saline solution, dried, and immersed in the fungal solution for 3 min. At least two different suspensions of the fungal conidia were used. The suspension concentration ranged between 10^2^ and 10^8^ cfu/ml. To each tested fungi strain, the control group treated with a saline solution was conducted simultaneously. After the immersion, ticks were transferred to the sterile containers with tissue paper moistened with sterile distilled water to keep 80% of the relative humidity and kept in the darkness at a temperature of 23 °C. Mortality observations were made daily over 3 weeks, and the paralysis of ticks (straightened legs, no response to CO_2_ stimuli) was recognized as the lethal effect.

### Statistical analyses

The data on the effects of the fungi on ticks was presented as a dosage causing the death of 50% of the population (LC_50_). The LC_50_ values were calculated using the Finney (1952) probit analysis method with the LC_50_/LD_50_ calculator. The tool is designed to calculate the dosages with the Abbot’s correction.

## Results and discussion

**Fig. 1:**
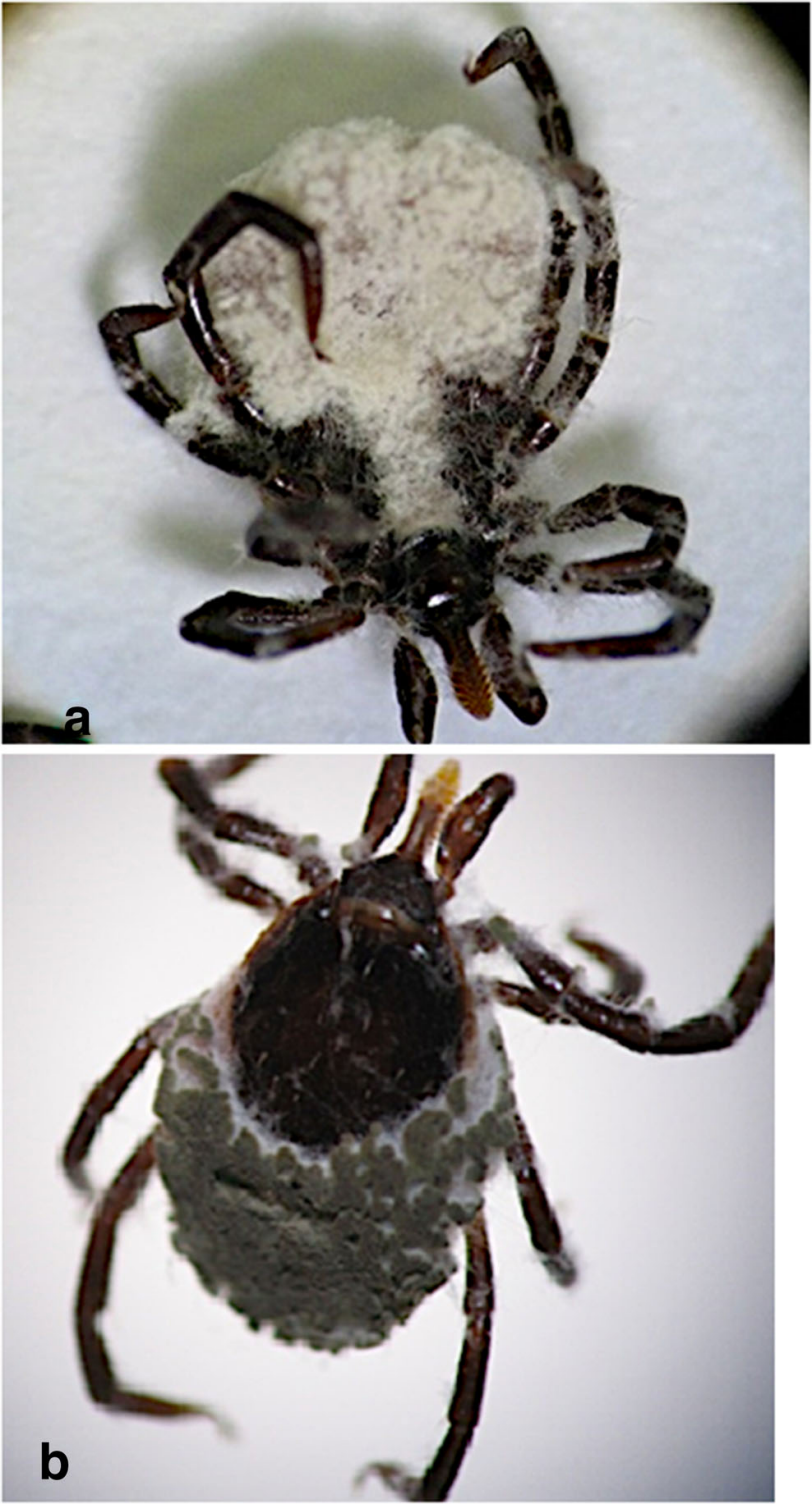
Fungal mycelia of *M. anisopliae* (Met52) growth on the cuticle of *I. ricinus* female. **A** Ventral side. **b** Dorsal side

Obtained fungi strains, with potential entomopathogenic properties from soil, were classified into 4 species: *Metarhizium anisopliae*, *Metarhizium robertsii*, *Isaria fumosorosea*, and *Beauveria bassiana*. The results of bioassay showed the difference in the potential of tick control, between the tested fungi strains and the tick species (Table 1; Fig. 1). However, the majority of our environmental isolates of fungi and commercial strains showed potential efficacy against both *Ixodes ricinus* and *Dermacentor reticulatus*. The observed mortality of unfed ticks was up to 100% using both commercial strains (Botanigard, MET52) as well as three environmental strains: *B. bassiana* LO37(1), *M. anisopliae* LO10(1), and *M. anisopliae* LO4(1). We found *D. reticulatus* to be less susceptible to entomopathogenic fungi than *I. ricinus*. Males of both tick species were much less susceptible compared to females. The most effective against both tick species was *M. anisopliae* LO4(1) with LC_50_ for *I. ricinus* of 2.6 × 10^3^ cfu/ml (females) to 5.2 × 10^4^ cfu/ml (males) and for *D. reticulatus* of 1.0 × 10^4^ cfu/ml (females) to 5.7 × 10^5^ cfu/ml (males). The efficacy of environmental fungi strains, including *M. anisopliae*, was also confirmed in the case of immature and engorged *I. ricinus*. Hartelt et al. (2008) observed limiting the number of nymphs to 80% after 30 days. Pirali-Kheirabadi et al. (2016) obtained up to 100% mortality against populations of engorged females of *I. ricinus* after using *M. anisopliae* IRAN 437C at a concentration of 2.4 × 10^7^ spores/ml.

**Table 1:** Lethal concentrations (LC_50_) of *Ixodes ricinus* and *Dermacentor reticulatus* infected with different fungal strains

Strain	Germination (%)	LC_50_ (cfu/ml)			
		*I. ricinus* (♀)	*I. ricinus* (♂)	*D. reticulatus* (♀)	*D. reticulatus* (♂)
Botanigard	97	6.8 × 10^3^	3.3 × 10^6^	5.9 × 10^5^	5.9 × 10^5^
MET52	95	1.6 × 10^6^	9.5 × 10^4^	2.0 × 10^6^	3.9 × 10^5^
*I. fumosorosea* LO34(3)	96	1.2 × 10^8^	1.9 × 10^9^	1.0 × 10^5^	1.5 × 10^14^
*M. robertsii* LO26(2)	92	3.9 × 10^6^	2.7 × 10^5^	3.3 × 10^6^	4.6 × 10^6^
*B. bassiana* LO37(1)	97	5.6 × 10^6^	3.4 × 10^6^	1.5 × 10^7^	5.6 × 10^7^
*M. anisopliae* LO10(1)	91	8.7 × 10^3^	3.8 × 10^3^	5.5 × 10^4^	4.4 × 10^7^
*M. anisopliae* LO52(1)	92	2.9 × 10^7^	2.9 × 10^8^	1.2 × 10^7^	6.0 × 10^7^
*M. anisopliae* LO52(2)	95	1.4 × 10^10^	n.e.	5.0 × 10^6^	n.e.
*M. anisopliae* LO4(1)	95	2.6 × 10^3^	5.2 × 10^4^	1.0 × 10^4^	5.7 × 10^5^
*B. bassiana* IGM	90	5.2 × 10^7^	9.8 × 10^8^	8.1 × 10^8^	5.6 × 10^6^
*M. anisopliae*LO47(3)	98	1.0 × 10^7^	1.2 × 10^7^	n.e.	n.e.

*n.e.* not effective

In our study, the tick of both species was less susceptible to *B. bassiana* comparing to *M. anisopliae*. *B. bassiana* is also effective against *I. scapularis* and *Rhipicephalus sanguineus* and less effective against *D. variabilis* (Kirkland et al. 2004). Our research found *I. fumosorosea* as the least effective fungi species against ticks. Low mortality with using *I. fumosorosea* was also observed for *R. sanguineus* larvae (< 10% mortality after 7 days) and *Rhipicephalus microplus* larvae (5.5% mortality after 10 days) (Samish et al. 2001; Angelo et al. 2012). Thus, *I. fumosorosea* turned out to be the least useful in tick control.

Among commercial strains, Botanigard proved to be more effective than MET52 with LC_50_ ranging between 6.8 × 10^3^ cfu/ml for *I. ricinus* females (Botanigard) and 2.0 × 10^6^ cfu/ml for *D. reticulatus* females (MET52). The virulence of Botanigard was also confirmed for the ticks of *Hyalomma lusitanicum*, and *Amblyomma americanum* limiting the average number of tick species in the environment by 90%, and on the host (*Oryctolagus cuniculus*) up to 80% (Cradock and Needham 2011; González et al. 2016).

Our research shows that several fungi strains can be considered as candidates for the biological control of *I. ricinus* and *D. reticulatus* ticks. However, further investigations, including in vivo tests, are required. Among local environmental entomopathogenic fungi tested in our study, the most promising for the biological control of *I. ricinus* and *D. reticulatus* turned out to be *M. anisopliae* strain LO4(1) as well as commercial products Botanigard and MET52.
